# High-performance optoelectronic and thermoelectric properties of transparent conductors based on Tl_2_O_3_ under pressure

**DOI:** 10.1038/s41598-024-58657-9

**Published:** 2024-04-22

**Authors:** H. A. Rahnamaye Aliabad, A. Asadpour Arzefooni, Seyede Zeinab Sadati, Evren Görkem Özdemir, P. Khosrojerdi

**Affiliations:** 1 Department of Physics, Hakim Sabzevari University, Sabzevar, 96179–76487 Iran; 2 Department of Physics, Faculty of Science, Gazi University, Teknikokullar, 06560 Ankara, Turkey

**Keywords:** Tl_2_O_3_, Optoelectronic and thermoelectric properties, Pressure, DFT, Electronic properties and materials, Optics and photonics, Electronic structure

## Abstract

In this work, the full-potential linearized augmented plane wave method (FP- LAPW) and the modified Becke-Johnson (mBJ) functional with spin–orbit (SO) coupling are used the obtain the structural, optoelectronic and thermoelectric properties of Tl_2_O_3_ under pressure. The results show that Tl_2_O_3_, as transparent conducting oxide (TCO), is a direct bandgap semiconductor with a band gap of 1.23 eV. The band gap value and the effective mass of electrons increases by increasing pressure. Density of state spectra reveal that the nature of electrons in Tl-6s state in the bottom of conduction band, like free electrons in *s* state, is responsible for the conducting behavior of Tl_2_O_3_. A blue shift is observed in optical spectra such as electron energy loss and absorption spectra with an increase in pressure. Obtained dielectric constants under pressure are inversely proportional to the band gap value according to Penn model. The effects of pressure on thermometric properties are also explored. The hydrostatic pressure increases Seebeck coefficient, while it decreases thermal conductivity that is an effective way to the enhancement of the thermoelectric efficiency of TCOs. A figure of merit (ZT) of 0.98 in p-type Tl_2_O_3_ is achieved that is desirable for using in thermoelectric devices.

## Introduction

Transparent thermoelectric materials have been extensively investigated in clean and renewable energy sources. Transparent conducting oxides (TCOs) such as Tl_2_O_3_, In_2_O_3_, TiO_2_, SnO_2_, and ZnO are used in liquid crystal and touch-screen displayers, photovoltaic cells, and thermoelectric devices due to their transparency in the visible light range and attractive electronic properties^1–4^. The computed hole mobility for Li_3_Sb is quite exceptional and comparable with the electron mobility in the best n-type TCOs^5^.

Among the TCOs with bixbyite structure, Tl_2_O_3_ is a degenerate n-type semiconductor with high conductivity and very low resistivity of about 10^–4^ Ω cm^–1^^6^. In Tl_2_O_3_ transparent conductor^7–10^, the carrier concentration changes significantly with oxygen partial pressure^10–13^ and semiconducting nature is observed by defect-induced in the crystal structure of compound. The metallic behavior of Tl_2_O_3_ probably originates from oxygen vacancy defects^9^.

The experimental and theoretical studies show that optical band gap of Tl_2_O_3_ change from 1.40 to 2.75 eV^8,11,14^ and 0.33 to 0.6 eV^7,14^, respectively. This difference in band gap values originate from additional electrons introduced into the material by some n-type defects, and self-doping due to the oxygen vacancy formation^7,9^. The carrier concentration of Tl_2_O_3_ increase by defects and the oxygen vacancies and reach 7.6 × 10^20^ cm^–3^^9^. Thallium-doping with the electron configuration of [Xe] 6s^2^4f^14^5d^10^6p^1^ is suitable way to reduces the band gap and increase the carrier mobility of In_2_O_3_^15^.

In the new TCOs, such as ZnSb_2_O_6_ (Ga- doping)^16^ and Ga_2_O_3_ (Si- doping)^17^ compounds, the band gap values for the pure and doped-TCOs increases from 3.38 to 3.56 eV and 4.80 to 4.99 eV, respectively. The widening of the band gap is due to the Burstein-Moss (B. M.) shift.

The band gap value can be altered by change in the carrier concentrations from *E*_g_ to $\mathop {{\acute{E}}_{g} }\limits$ as the Burstein-Moss (B. M.) effect^7^. The B. M. shifts of the band gap is due to the filling the bottom of the conduction band. The band gap value is given by Ref.^18^:

1 $$\mathop {{\acute{E}}_{g} }\limits {\text{ = E}}_{{\text{g}}} { + }\Delta {\text{E}}_{{\text{g}}}^{{{\text{BM}}}} ,$$

where $\Delta {\text{E}}_{{\text{g}}}^{{{\text{BM}}}}$ is the Burstein–Moss energy shift given by:

2 $$\Delta E_{g}^{BM} = \frac{{\hslash }^{2}}{{2m_{vc}^{*} }}(3\pi^{2} n_{e} )^{2/3}$$

Optical transitions of carriers are interpreted by considering the Burstein-Moss (B. M.) shift in the conduction band^13^. Optical reflectivity spectra show that there is a strong reflectance in the near-infrared region (1300–1500 nm)^14^. The B. M. mechanism is observed in transparent conductors like Tl_2_O_3_ and narrow band gap materials for using in solar cells and high-temperature thallium oxide-based superconductors^19^. Narrow band gap materials are typically good thermoelectric materials^20^. However, development of transparent conductors with high thermoelectric efficiencies is one of effective ways to produce electricity without losing their transparency. Narrow electronic states play a key role in the TCOs for thermoelectric applications.

The structural, optical, and thermoelectric properties are sensitive to the applied pressure^21^. On the other hand, the effect of pressure has been a powerful method for studying the optoelectronic and thermoelectric properties of materials in the past few decades. The thermoelectric efficiency of a material can be improved by applying pressure^22–29^.

The efficiency of thermoelectric materials is defined by the figure of merit, *ZT* = *S*^*2*^*σT/κ*, where S, *σ*, *T* and *κ* are the Seebeck coefficient, electrical conductivity, absolute temperature, and total thermal conductivity, respectively. It is clear that high *ZT* value requires high σ, low κ, and relatively high S. The electronic structure of materials is important for the reach high *ZT* values^22^.

The experimental results show that Tl_2_O_3_ has a cubic bixbyite crystal structure with $Ia\overline{3 }$ space group and lattice constant of a = 10.5344 Å^30^. Gomis et al. have studied the structural properties of Tl_2_O_3_ under high pressures^31^. Their results indicate that the crystal structure of Tl_2_O_3_ is cubic bixbyite to 22.0 GPa. At room temperature, the amorphous phase of Tl_2_O_3_ is predicted above 22 GPa by the theoretical analysis of the elastic properties^31^.

In the present work, we have investigated the structural, optoelectronic, and thermoelectric transport properties of Tl_2_O_3_ under high pressures up to 22.0 GPa by using the DFT as implemented in the Wien2k^32^ and BoltzTrap codes^33^. The method of calculations is described in Sect. "Computational details". The optoelectronic and thermoelectric results are presented and discussed in Sect. "Results and discussions" and conclusions is summarized in Sect. “Conclusion”.

## Computational details

Pressure -dependent optoelectronic and transport properties of Tl_2_O_3_ were calculated by the full-potential linearized augmented plane wave method (FP- LAPW). For calculation of the exchange–correlation potentials, the modified Becke-Johnson (mBJ) functionals^34–38^ with spin–orbit coupling are used as implemented in the Wien2k package. The electronic wave functions are expanded by the plane-wave cutoff value of K_max_ × R_MT_ = 7.0 in the interstitial region (R_MT_ is the smallest atomic muffin tin sphere radius). G_max_, the magnitude of the largest vector in the Fourier expansion of charge density, is selected about 12 (Ry)^1/2^. The self-consistent field (SCF) calculations are carried out with 600, 1800, and 12,000 k-points for structural, optoelectronic, and thermoelectric calculations, respectively.

The Boltzmann transport equation by employment of the rigid band approximation is used to obtain the thermoelectric properties. In this approximation, a shift of the Fermi level is equivalent to the doping of the compound. The Fermi level is defined as: ${E}_{f}=\frac{{\hslash }^{2}}{2{m}^{*}}{(3{\pi }^{2}n)}^{2/3}$, where *n* and ${m}^{*}$ are carrier concentration and the effective mass, respectively. The thermoelectric calculations are performed with *n* = 1 × 10^18^ to 1 × 10^21^ cm^–3^ carrier concentrations at 50 to 800 K. To obtain the best thermoelectric efficiency of Tl_2_O_3_ under different pressures and carrier concentrations, we have compared the obtained results under various carrier concentration to each other. It is found that *n* = 1 × 10^18^ cm^–3^ is an effective carrier concentration and it gives us the optimized achievement. Therefore, we have just presented the thermoelectric results with *n* = 1 × 10^18^ cm^–3^. Here, the relaxation time is considered as a constant, and we have used the following relations^23^:

3 $${\sigma }_{\alpha \beta }\left(\varepsilon \right)=\frac{1}{N}{\sum }_{i,k}{\sigma }_{\alpha \beta }\left(i,k\right)\frac{\delta \left(\varepsilon -{\varepsilon }_{i,k}\right)}{\delta \left(\varepsilon \right)},$$

4 $${\nu }_{\alpha \beta }(T;\mu )=\frac{1}{eT\Omega }\int {\sigma }_{\alpha \beta }\left(\varepsilon \right)\left(\varepsilon -\mu \right)\left\{-\frac{\partial {f}_{\mu }\left(T;\varepsilon \right)}{\partial \varepsilon }\right\}d\varepsilon ,$$

5 $${S}_{i,j}={({\sigma }^{-1})}_{\alpha i}{\nu }_{\alpha j},$$

6 $${\kappa }_{\alpha \beta }^{0}\left(T;\mu \right)=\frac{1}{{e}^{2}T\Omega }\int {\sigma }_{\alpha \beta }\left(\varepsilon \right){\left(\varepsilon -\mu \right)}^{2}\left\{-\frac{\partial {f}_{\mu }\left(T;\varepsilon \right)}{\partial \varepsilon }\right\}d\varepsilon ,$$

where *μ*, *f*_*μ*_ and *κ*^*0*^ are chemical potential, Fermi-Dirac distribution function and the electronic part of thermal conductivity (*κ*^*0*^ = *κ*_*e*_), respectively. The total thermal conductivity is composed of two electronic *κ*_*e*_ and lattice *κ*_*l*_ thermal conductivities (*κ* = *κ*_*e*_ + *κ*_*l*_). A direct method for study of lattice thermal conductivity* κ*_*l*_, is molecular dynamic simulation. A long simulation time need to calculate the *κ*_*l*_ and prediction of *κ*_*l*_ for Tl_2_O_3_ with the large unit cell is very expensive and it is impossible by Wien2k and BoltzTrap codes. On the other hand, there is not experimental evidence for *κ*_*l*_ of Tl_2_O_3_ and compared to the previously reported results for Tl_2_O show that *κ*_*l*_ of Tl_2_O is below 1 W/mK^39^ and it has a negligible contribution from the total thermal conductivity *κ*. Nevertheless, here we have calculated the electronic part of thermal conductivity* κ*_*e*_.

For a semiconductor with a narrow band gap, the dependence of the Seebeck coefficient (S) on the effective mass of carriers is calculated as follows:

7 $$S=\frac{8{\pi }^{2}{k}_{B}^{2}}{3e{h}^{2}}{({m}_{1}^{*}{m}_{2}^{*}{m}_{3}^{*})}^{1/3}{N}_{v}^{2/3}T{(\frac{\pi }{3n})}^{2/3},$$

8 $${m}_{i}^{*}=\frac{\int {e}^{-\frac{dE\left(k\right)}{{k}_{B}T}}{m}_{b}^{*}\left(k\right)d(k)}{\int {e}^{-dE(k)/{k}_{B}T}dk},$$

9 $${m}_{b}^{*}=\frac{{\hslash }^{2}}{\frac{{\partial }^{2}E(k)}{\partial {k}^{2}}},$$

where *ℏ*, *N*_*v*_, ${m}_{i}^{*}$ and ${m}_{b}^{*}$ are the Planck constant, band degeneracy, the mass components along three perpendicular directions (*i* = *x, y, z*) and band mass, respectively^40^.

For study of optical spectra, the complex dielectric function is calculated. Dielectric function is the linear response of a system to electromagnetic waves and can be described by both the real *ε*_1_(ω) and imaginary *ε*_2_(ω) parts which are calculated as following relations^41^:

10 $${\varepsilon }^{\alpha \beta }\left(\omega \right)={\varepsilon }_{1}^{\alpha \beta }\left(\omega \right)+i{\varepsilon }_{2}^{\alpha \beta }\left(\omega \right),$$

11 $${\varepsilon }_{2}^{\alpha \beta }\left(\omega \right)=\frac{{\hslash }^{2}{e}^{2}}{\pi {m}^{2}{\omega }^{2}}\sum_{c\text{,}v}\int dk\langle {c}_{k}|{p}^{\alpha }|{v}_{k}\rangle \langle {v}_{k}|{p}^{\beta }|{c}_{k}\rangle \delta \left({\varepsilon }_{ck}-{\varepsilon }_{vk}-\omega \right),$$

12 $$\varepsilon_{1}^{\alpha \beta } \left( \omega \right) = \delta_{\alpha \beta } + \frac{2}{\pi }P \int \limits_{0}^{\infty } \frac{{\omega Im\mathop {{\acute{\varepsilon}}_{{\alpha \beta \left( {\mathop {\acute{\omega}} } \right)}} }}}{{\mathop {\acute{\omega}} ^{2} - \omega^{2} }}d\mathop {\acute{\omega}} ,$$

13 $${\alpha }_{\alpha \alpha }\left(\omega \right)=\frac{\sqrt{2 }\omega }{c}\sqrt{\left|{\varepsilon }_{\alpha \alpha }\right|-{Re\varepsilon }_{\alpha \alpha }\left(\omega \right),}$$

14 $${Eloss}_{\alpha \alpha }\left(\omega \right)=Im\left(\frac{-1}{{\varepsilon }_{\alpha \alpha }\left(\omega \right)}\right).$$

## Results and discussions

### Optoelectronic results

**Figure 1:**
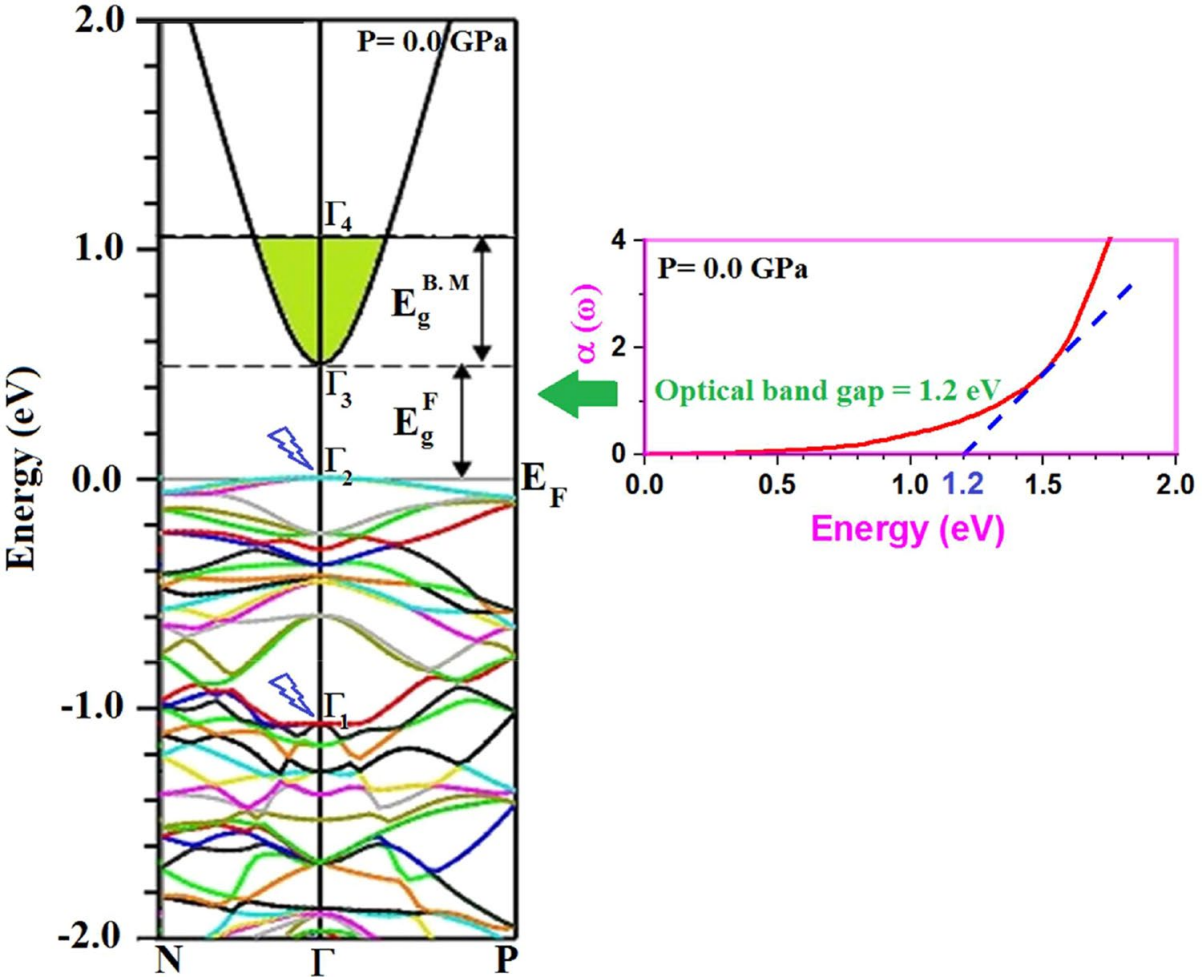
Calculated band structure of Tl_2_O_3_ and band gap engineering by *mBJ* at zero pressure. Obtained band gap is compared with the calculated absorption spectra at zero pressure.

The electronic band structure (B. S) of Tl_2_O_3_ was calculated using the mBJ and mBJ + SO potentials and the obtained B. S spectrum at zero pressure is shown in Fig. 1 by mBJ. A direct band gap ${E}_{g}^{F}$ of 0.49 eV is appeared between top of the valence band Γ_2_ and bottom of the conduction band Γ_3_. The experimental results show that there is a Bursntein-Moss shift ${E}_{g}^{B. M}$ about 0*.*74 eV in Tl_2_O_3_ compound between Γ_3_ and Γ_4_ points^13^. Therefore, widening of the band gap is about 1.23 eV. On the other hand, the optical band gap value at zero pressure was obtained by optical absorption calculations and compared with the electronic band structure. The results of the electrical and optical calculations for pure Tl_2_O_3_ and other TCOs have been summarized in Table 1. Obtained band gap value is in close agreement with the experiment. The bottom of the conduction band exhibit Tl-6s dangle band around the Γ point. The presence of parabolic curve, like free electrons in *s* state, justify the conductor behavior of Tl_2_O_3_.

**Table 1:** Obtained optoelectronic and thermoelectric properties (at 300 K) of Tl_2_O_3_ and compared with the other TCO materials.

TCOs	${{\text{E}}}_{{\text{g}}}^{{\text{DFT}}}$(eV)	${{\text{E}}}_{{\text{g}}}^{{\text{Exp}}.}$(eV)	ε(0)	ZT	S (μV/K)
Tl_2_O_3_	1.23, 1.1^a^	1.4^b^, 1.6^c^	4.80	0.82(n-type), 0.98 (p-type)	− 292 (n-type), 775 (p-type)
In_2_O_3_	0.99^d^, 1.2^e^	2.61^d^	4.73(DFT)^d^, 4.0 (Exp.)^d^	–	320^f^
Tl_2_O	0.93^g^,1.62^h^	–	–	0.96^ h^, 0.94^ h^	–
HgO	1.17^g^, 1.28^i^	2.2^c^, 1.9^c^	–	0.58^i^	–
In_1.5_Co_0.5_O_3_	1.3^f^, 0.8^f^	–	4.41^f^	–	–
In_1.5_La_0.5_O_3_	3.03^j^	4.03^j^	3.12^j^	–	–
SnO_2_	1.76^k^	3.41^k^, 3.87^l^			255^l^
α-Ga_2_O_3_	2.86^k^	4.9^k^	–	–	–
β-Ga_2_O_3_	2.29^k^	4.61^k^	4.37^m^	–	290(Exp_)_^f^, 341 (DFT)^f^

^a^Ref.^7^, ^b^Ref.^8^, ^c^Ref.^9^, ^d^Ref.^38^, ^e^Ref.^36^, ^f^ Ref.^41^, ^g^ Ref.^39^, ^h^Ref.^42^, ^i^Ref.^43^, ^j^Ref.^35^, ^k^Ref.^44^, ^l^Ref.^45^, ^m^Ref.^46^.

**Figure 2:**
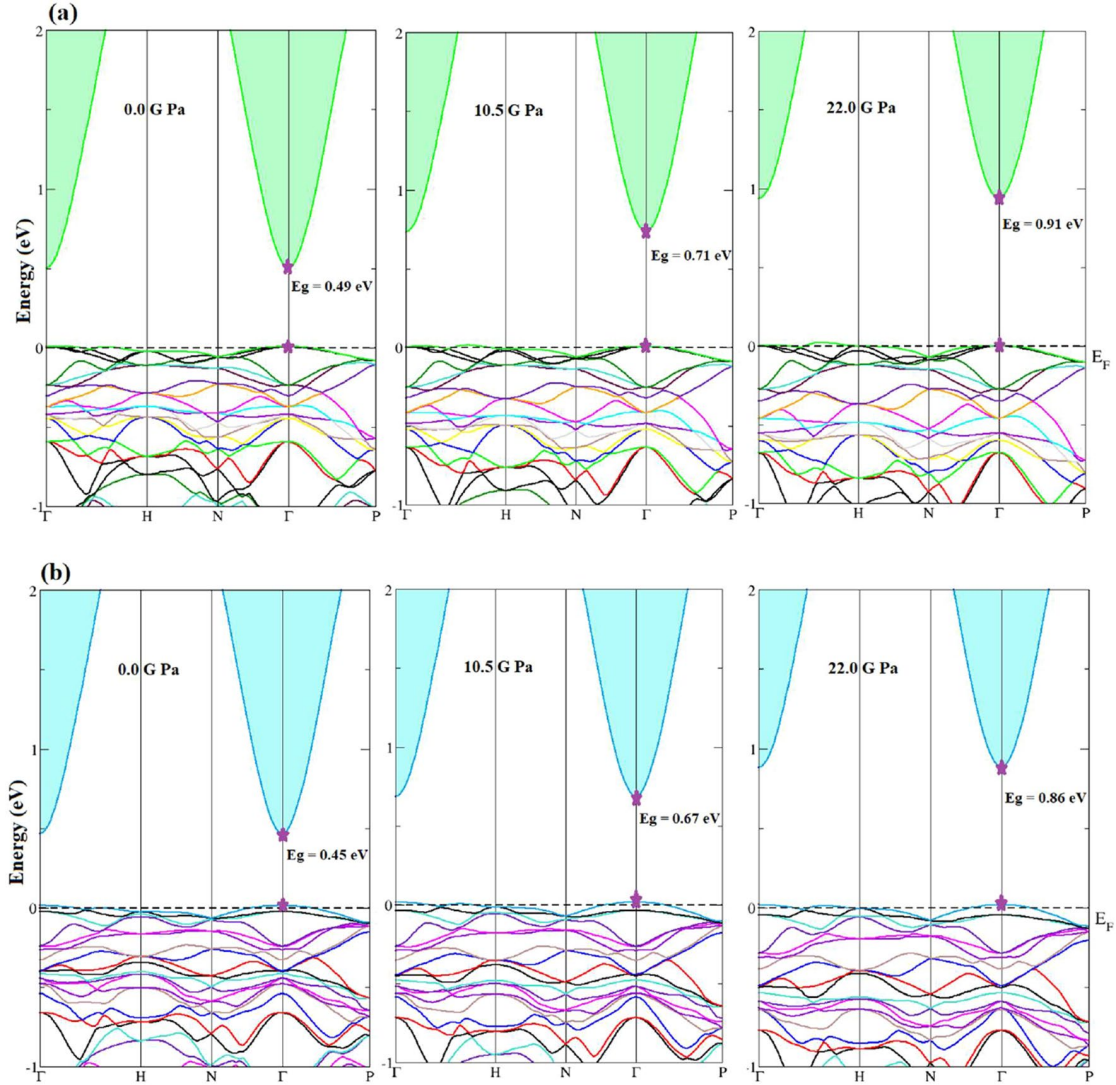
Calculated band structures of Tl_2_O_3_ under pressure by (**a**) *mBJ* and (**b**) *mBJ* + *SO*.

**Figure 3:**
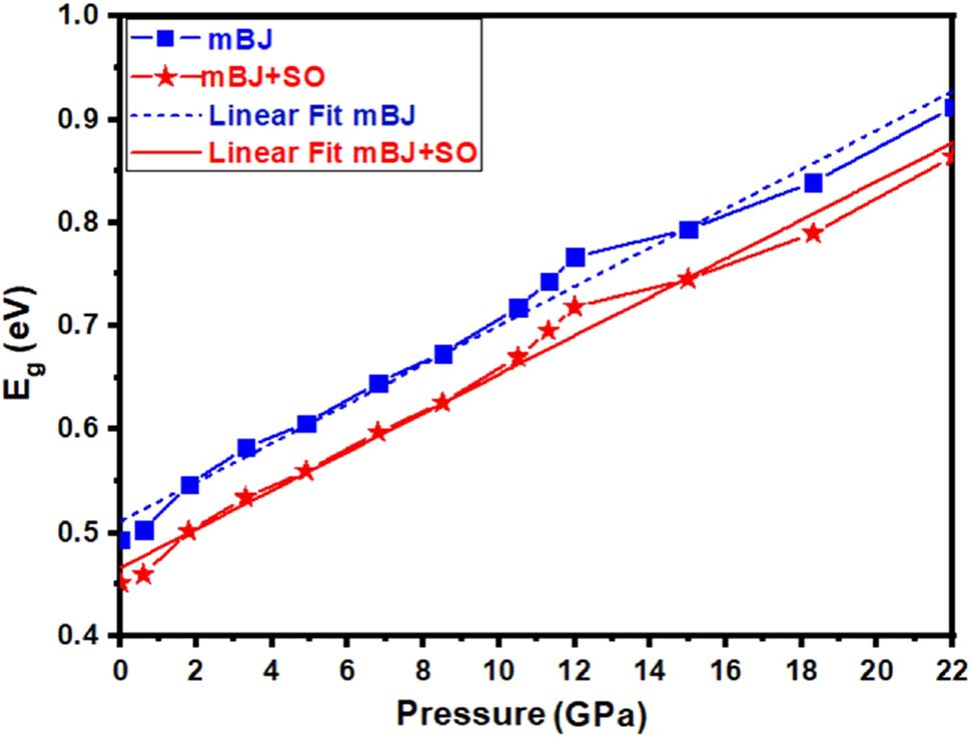
The variation of the band gap value with respect to the pressure for Tl_2_O_3_.

The calculated band structure of the Tl_2_O_3_ under pressure is plotted in Fig. 2. The band gap’s nature is direct at *Γ* direction under all pressures. Obtained band gap values at zero pressure are achieved 0.49 and 0.45 eV for mBJ and mBJ + SO, respectively, which are in close agreement with the band gaps reported by other (see Table 1)^8,9,35–39,42–46^. The variation of band gap values with pressures is shown in Fig. 3. It is observed that the band gap value linearly increases with pressure and band gap values by mBJ are more than mBJ + SO at all pressures. We have fitted two linear relations between band gap values and pressures as:

15 $${\text{E}}_{\text{g}}^{\text{mBJ}}\text{= 0.01891 P+ 0.5114}\text{; } {\text{E}}_{\text{g}}^{\text{mBJ+SO}}\text{= 0.01869 P+ 0.4665}.$$

**Figure 4:**
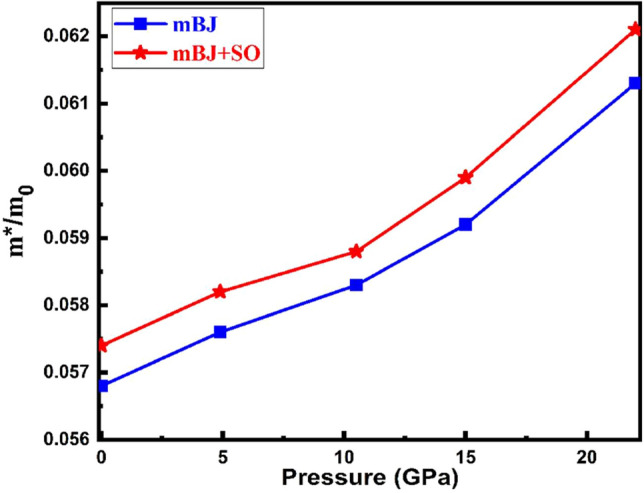
The variation of the effective mass of electrons with respect to the pressure for Tl_2_O_3_.

The results indicate that it is possible to control the band gap value and the other optoelectronic and thermoelectric properties by pressure. Nevertheless, we to predict the transport properties of electrons under applied pressure by the calculation of the effective mass of carries from the curvature of band structure plots. To clarify the effective mass of electrons under pressure, the curvature of electronic bands in the bottom of the conduction band are investigated at Γ → P direction by Eq. (9). As shown in Fig. 4, with increasing pressure, the effective mass of electrons is increased and the electron mobility decreases. It should be noted that by increasing pressure, the lattice constants and hence bond lengths decrease and the interaction between electrons and ions increases which results in the change the effective mass of electrons.

**Figure 5:**
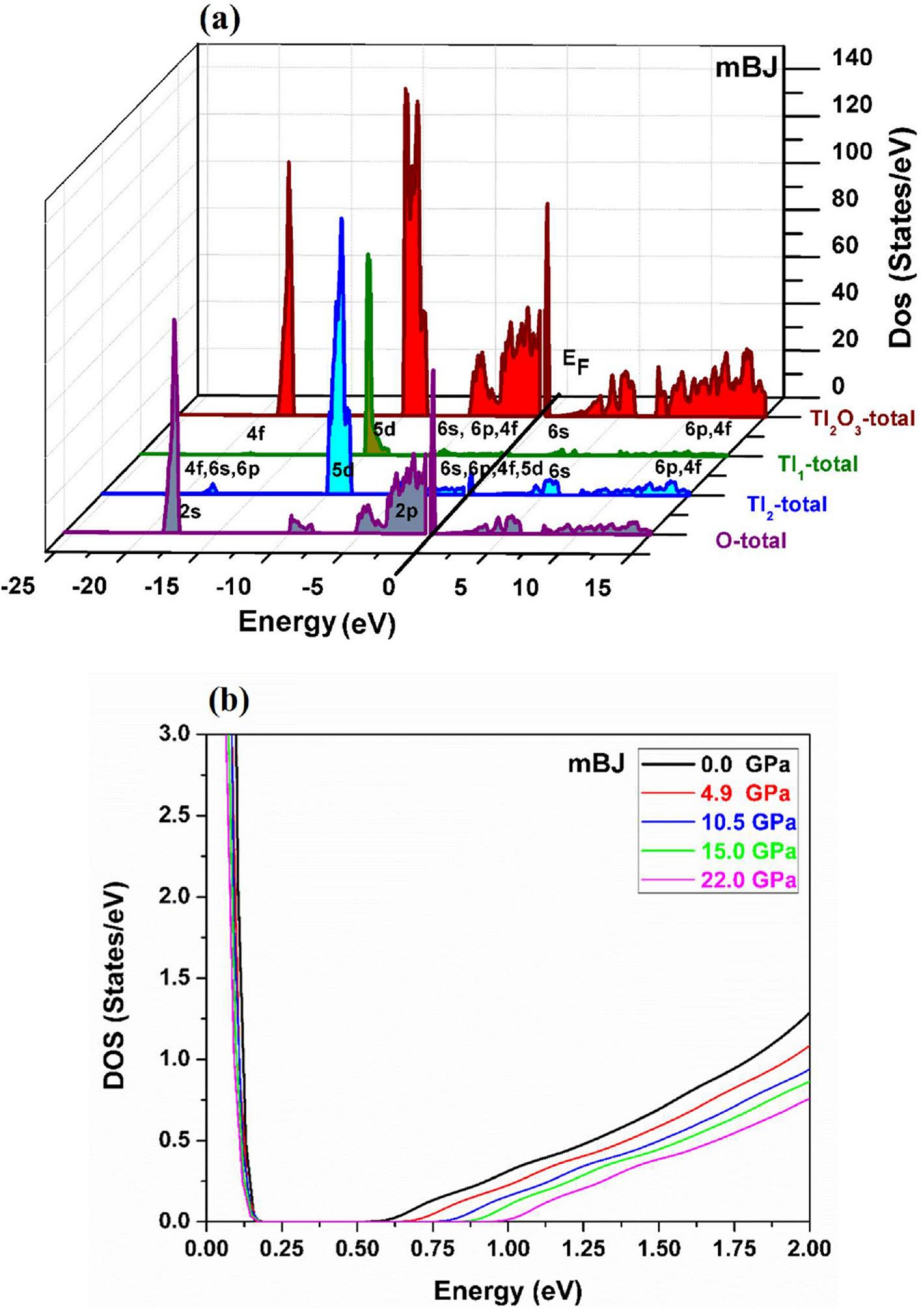
Density of states of Tl_2_O_3_ (**a**) at zero pressure and (**b**) different pressures by *mBJ*.

The calculated total and partial densities of states (DOSs) of Tl_2_O_3_ are presented in Fig. 5a at zero pressure. Three main regions are observed in DOS spectra. The first region is observed in − 19 to − 16 eV. It is a mixture of the O-2 s states with a few contributions of Tl-6 s, 4f, and 6p states. The second region is created in the − 10 to 0 eV near the Fermi level. It is considerably dominated by Tl-5d, 6 s, 6p, and 4f states and O-2p states while in the third region at the bottom of the conduction band, Tl-6 s and O-2p states play a key role in electronic behavior of Tl_2_O_3_.

Pressure dependence of the DOSs are plotted in Fig. 5b. It is clear that by increasing pressure, the valence band maximum is nearly constant while the conduction band minimum is shifted upward. It is found that the effect of hydrostatic pressure significantly shifts the Tl- 6s state in the bottom of the conduction band. Electronic results show that pressure tuning is an effective way to control the physical properties of TCOs.

**Figure 6:**
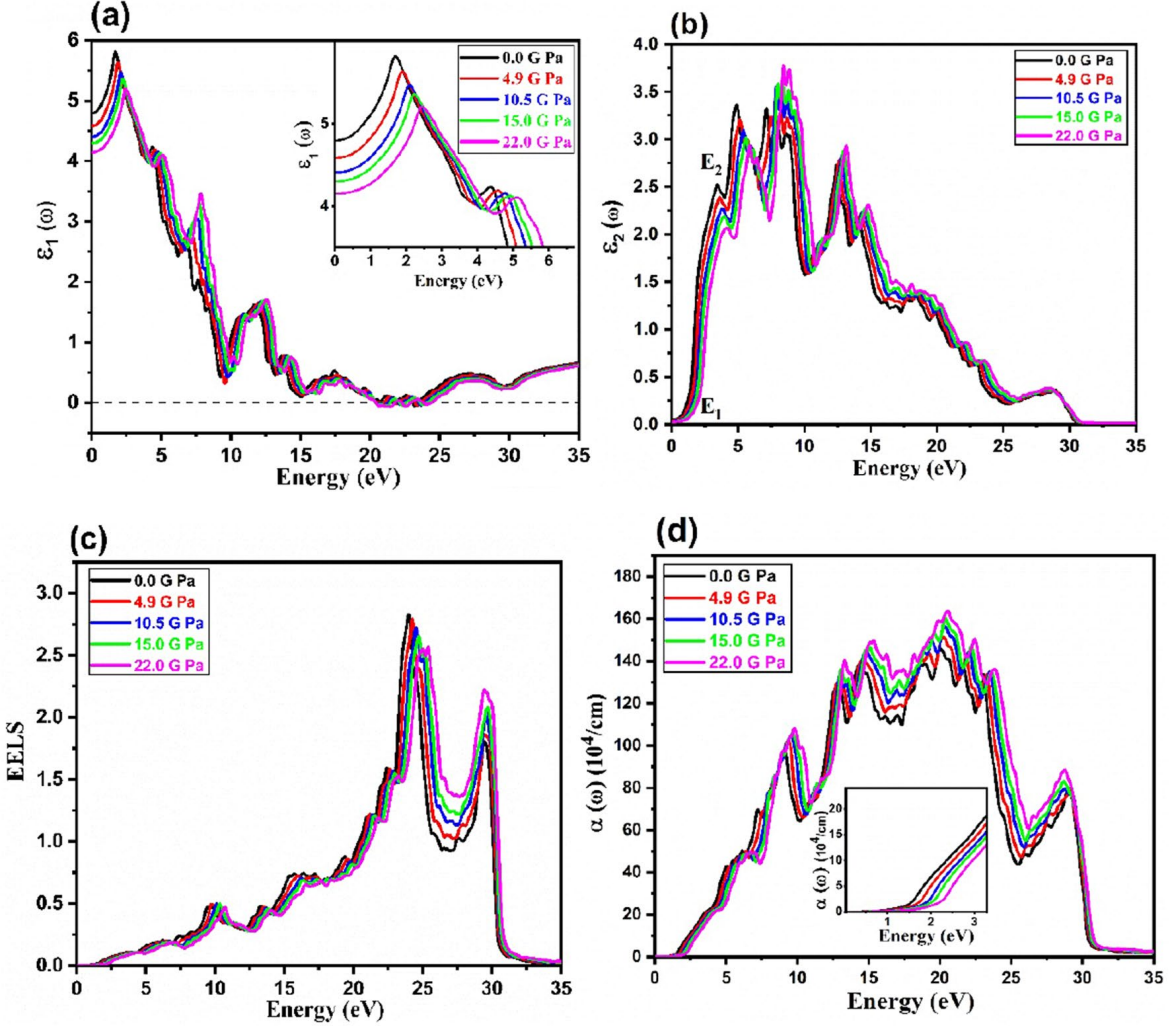
Calculated optical spectra for Tl_2_O_3_ at various pressures (**a**) the real part of dielectric function, (**b**) the imaginary part of dielectric function (**c**) the electron energy loss spectrum EELS and (**d**) Absorption.

In Fig. 6a and b, the real and imaginary parts of the dielectric function are plotted at different pressures. Obtained static dielectric constants at zero photon energy, *ε*_1_(0), are 4.8, 4.62, 4.4, 4.3, and 4.2 at 0.0, 4.9, 10.5, 15.0 and 22.0 GPa, respectively which compared with similar TCOs in Table 1^31,32,34^. It is also found that *ε*_1_(0) decreases with increasing pressure. Tl_2_O_3_ can be used in semiconductor devices because of the inverse relationship between the static dielectric constants and semiconductor band gap which verifies by the Penn model^47^ (${\upvarepsilon }_{1}\left(0\right)\approx 1+{(\frac{\mathrm{\hslash }{\upomega }_{{\text{p}}}}{{{\text{E}}}_{{\text{g}}}})}^{2}$).

By increasing pressure, the main peaks, corresponding to the different pressures, are shifted towards higher energies. For the first and second main peaks, the intensity of peaks decreases with an increase in pressure while for the other main peaks near to the zero value* ε*_1_(ω) the intensity of peaks increases with pressure. After some fluctuations, it reaches to zero value at 0.0 GPa in the range of 21.0 and 24.0 eV. It is clear that the negative value of *ε*_1_(ω) is almost appeared with increasing pressure. In this region, the material exhibits a high reflection with metallic nature. After 25 eV, we can to see the positive value of ε_1_(ω) and the overall trend of *ε*_1_(ω) remains the same at the high energy of incident photons under the effect of pressure.

As shown in Fig. 6b, the main peaks of the imaginary part of the dielectric function, *ε*_2_(ω), are corresponding to the optical transitions of electrons from the valence band to the conduction band around the *E*_F_. The optical band gap or threshold energy of *ε*_2_(ω) spectrum is shown as *E*_*1*_, correspond to direct inter-band transitions from Γ_2_ → Γ_4_ in Fig. 1. By increasing pressure, this value increase. The second strong peak *E*_*2*_ is corresponding to the optical transitions Γ_1_ → Γ_4_, as it is observed in the experimental absorption measurements^9^. Other main peaks are related to the electronic transitions from the deep electronic states in the valence band to the conduction band^35^.

The electron energy loss spectrum, EELS, is a useful optical spectrum for prediction of Plasmon energies. The electron density in the material plays a key role in the excitation of the plasmons. Figure 6c shows the calculated EELS spectra. EELS is zero at low energies blew the optical band gap without scattering of electrons. By enhancing the energy of incident photons, EELS increases slowly due to the inelastic scattering of electrons. EELS spectrum reaches to the maximum value then decreases. The maximum energy value of EELS corresponds to the Plasmon energy. Obtained Plasmon energies are 24, 24.2, 24.55, 24.69 and 25.38 eV at 0.0, 4.9, 10.5, 15.0 and 22 GPa, respectively. It is clear that the Plasmon energy is shifted to the higher value of EELS with increasing pressure.

In Fig. 6d, the pressure- dependent of absorption spectra of Tl_2_O_3_ are displayed at the energy range of 0–35 eV. By increasing pressure, the absorption value increase from 146.72 to 163.47 × 10^4^ cm^–1^ and a blue shift is observed towards higher values in the energy of incident photons correspond to the energy range of 15.32- 20.54 eV in the ultraviolet UV spectrum. As shown in the inset of Fig. 6d, there is not absorption for photons with energies lower than optical band gap and absorption start with an increase in photon energy. It is clear that optical band gap increase with an increase in pressure.

### Thermoelectric results

**Figure 7:**
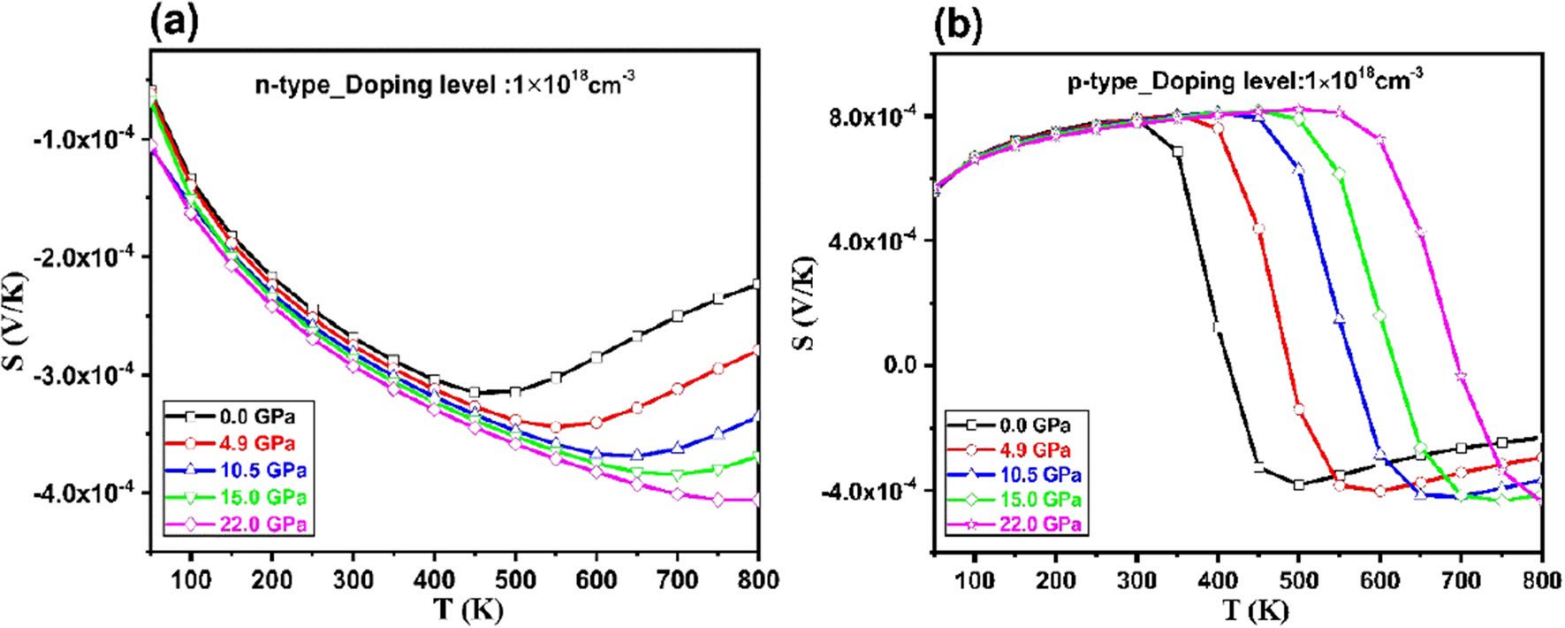
The variation of Seebeck coefficient with respect to the temperature and pressure at n = 1 × 10^18^ cm^–3^ carrier concentration for (**a**) *n-* and (**b**) *p* -types of Tl_2_O_3_.

As shown in Fig. 7, the Seebeck coefficient (*SC*) of Tl_2_O_3_ is calculated as a function of carrier concentration, pressure, and temperatures for both *n*- and *p*-types Tl_2_O_3._ The Seebeck coefficient is negative for n-type Tl_2_O_3,_ and it is positive for p-type. It is observed that the *SC* increase with increasing temperature then it decreases with temperature as defined in Eq. (7). The *SC* is directly proportional to the temperature and it is inversely proportional to the carrier concentration. Therefore, the *SC* increase with temperature at low temperature. On the other hand, at high temperatures, the carrier concentration *n* is thermally exited hence by increasing *n*, the *SC* decreases. Obtained *SC*, at room temperature (300 K) for *n*- type Tl_2_O_3_, are − 268, − 275, − 281, − 286 and − 292 μV/K at 0.0, 4.9, 10.5, 15.0 and 22 GPa, respectively. The results show that with increasing pressure, the *SC* for *n*-type one increases and it is almost similar for *p*-type one in the temperature range of 50–350 K.

As shown in Fig. 4 and defined in Eq. (7), by increasing pressure, the effective mass of electrons increases which results an increased *SC* of* n*- type Tl_2_O_3_. For *p*- type Tl_2_O_3_ with increase in pressure, the hole states in the valence band are nearly constant (Fig. 2) and hence its changes with pressure is almost small. It is also seen that the more effect of pressure on SC is at high temperatures.

**Figure 8:**
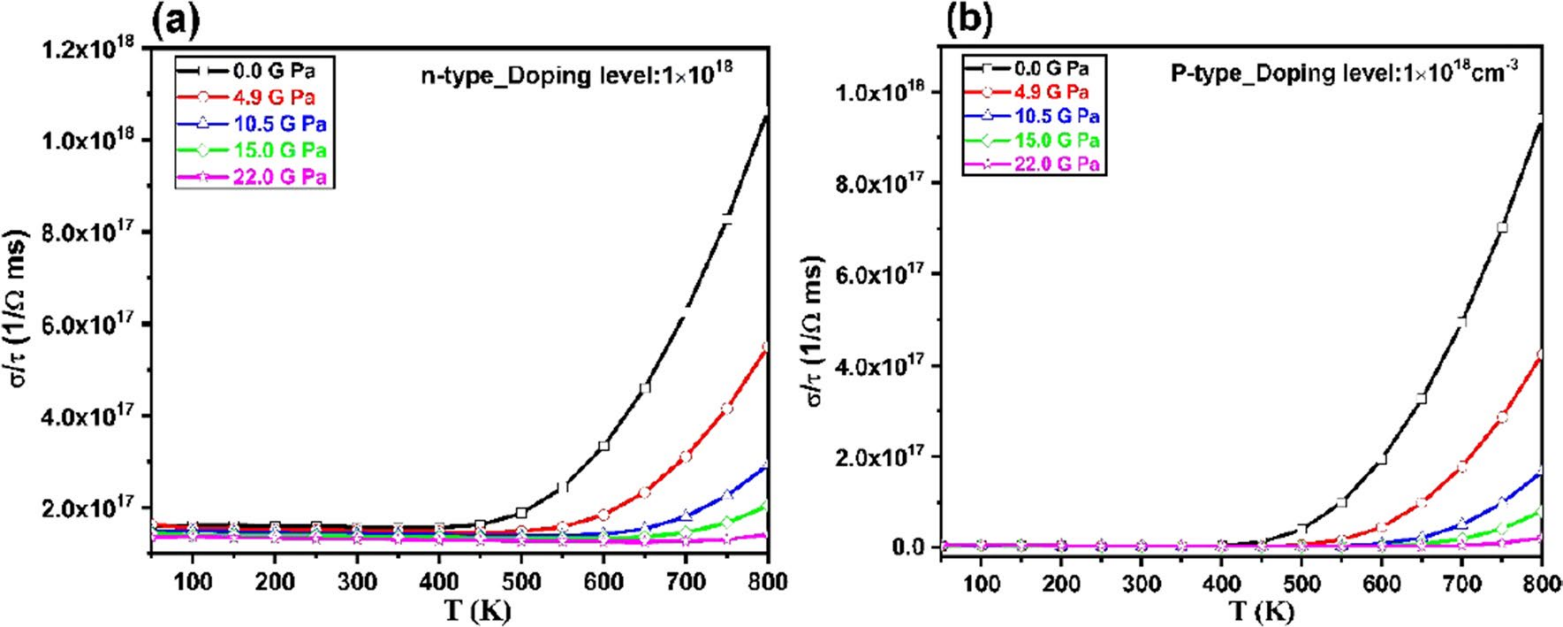
The variation of electrical conductivity with respect to the temperature and pressure at n = 1 × 10^18^ cm^–3^ carrier concentration for (**a**) *n-* and (**b**) *p* -types Tl_2_O_3_.

The calculated electrical conductivity (*σ/τ*) is plotted in Fig. 8. In this plot, the variation of *σ/τ* with *n* = 1 × 10^18^ cm^–3^ carrier concentration, temperature and pressure are studied. The electrical conductivity is almost constant at low temperature 450 K then it increases by increasing temperature and excitation of carrier concentration as following relation:

16 $$\sigma \, = \, ne\mu ,$$

where *μ* is the mobility. The figure shows that the electrical conductivity (*σ*/*τ*) remains almost constant with enhancing temperature at high pressure (22.0 GPa).

**Figure 9:**
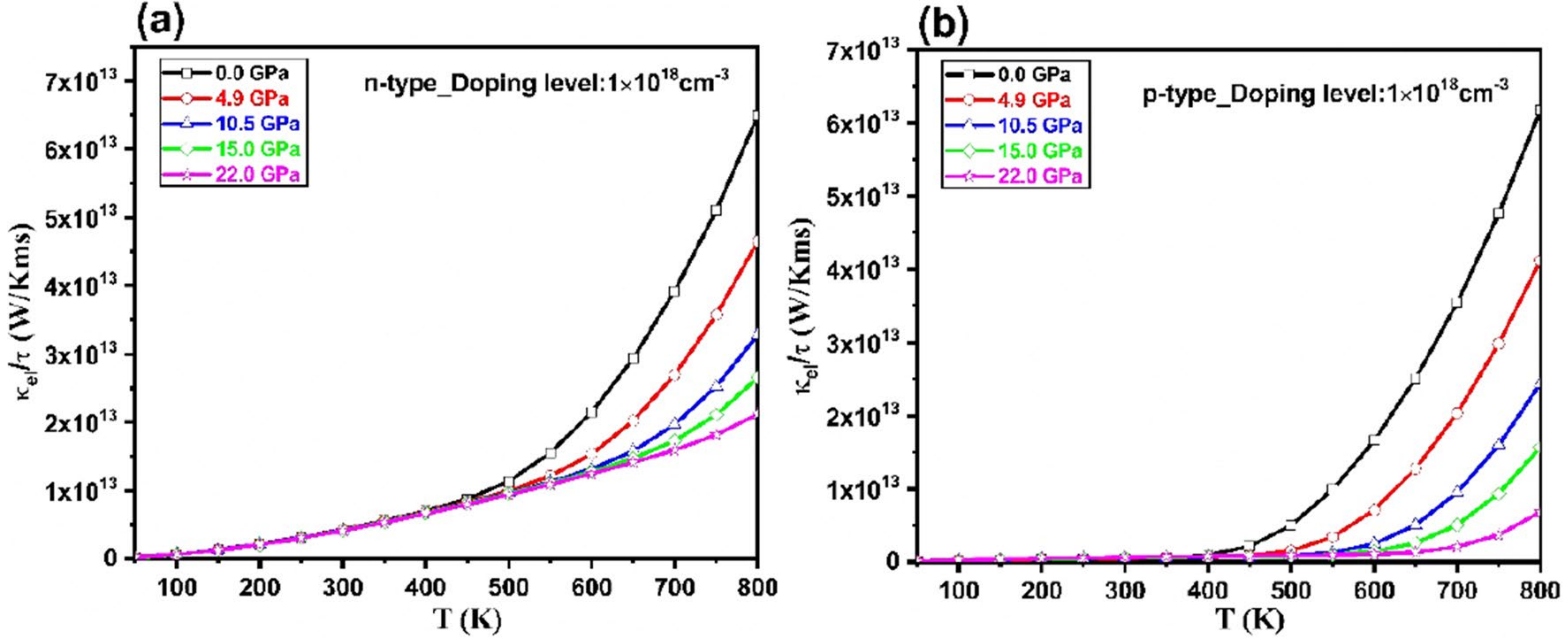
The variation of thermal conductivity with respect to the temperature and pressure at n = 1 × 10^18^ cm^–3^ carrier concentration for (**a**) *n-* and (**b**) *p* -types of Tl_2_O_3_.

The pressure and temperature dependences of electronic part of thermal conductivity (*κ*_e_*/τ*) are displayed in Fig. 9 for n- and p- type Tl_2_O_3_ at *n* = 1 × 10^18^ cm^-3^ carrier concentration. Electronic thermal conductivity depends upon carrier concentration and temperature, as follows:

17 $${k}_{e}=\frac{n{\pi }^{2}T{k}_{b}^{2}}{3{m}^{*}{v}_{e}}.$$

It is clear that by the increase in temperature, the electrical conductivity increases and it remains constant at all pressures at low temperatures. An increasing trend is observed just in n-type Tl_2_O_3_ which correspond the electronic states in the conduction band, with the pressure effect at low temperatures. At high temperatures, with the increase in pressure, the effective mass of carries increase, so the electronic thermal conductivity decreases. The maximum value of *κ*_*e*_/*τ* for n-type Tl_2_O_3_ is obtained about 6.5 × 10^13^ W/Kms at 800 K and zero pressure.

**Figure 10:**
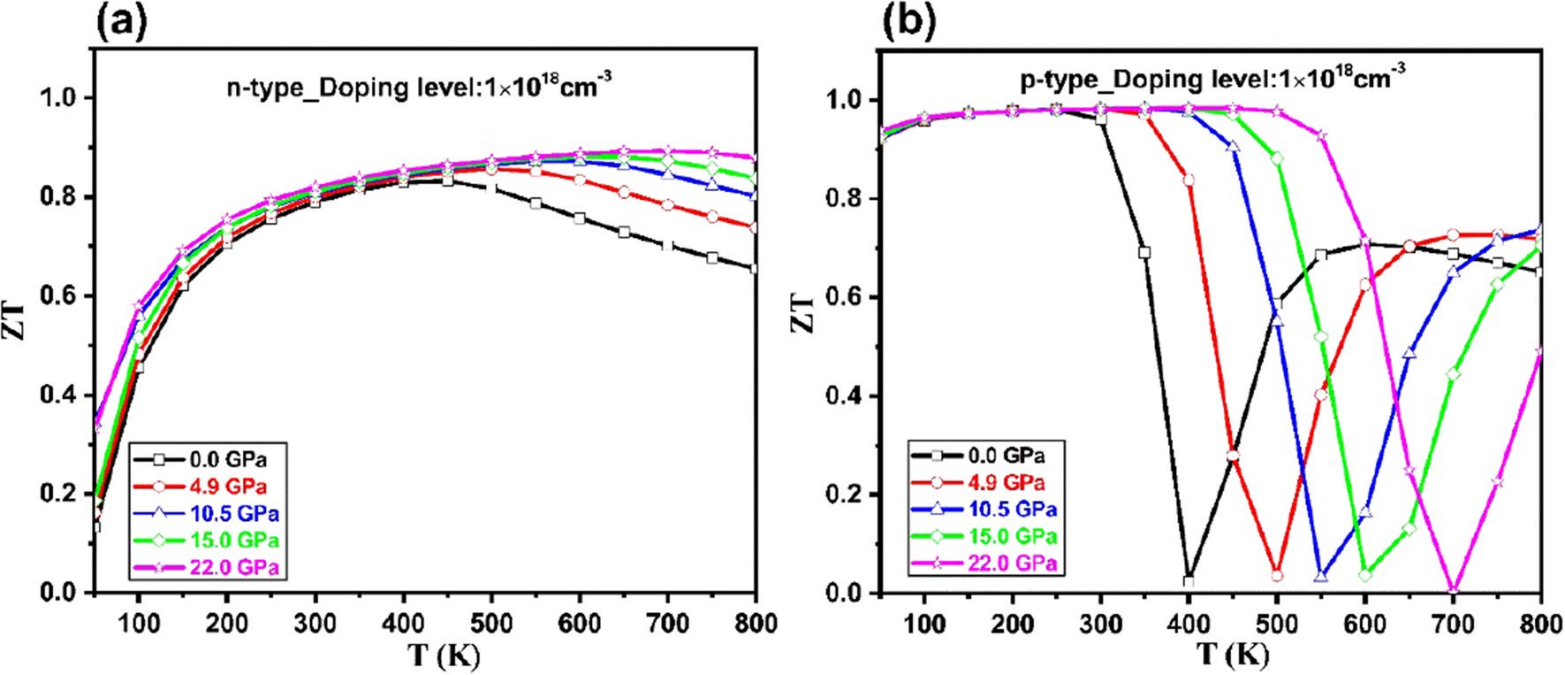
The variation of figure of merit (*ZT*) with respect to the temperature and pressure at n = 1 × 10^18^ cm^–3^ carrier concentration for (**a**) *n-* and (**b**) *p* -types of Tl_2_O_3_.

In Fig. 10, the variation of figure of merit (*ZT*) are shown with temperature and pressure at n = 1 × 10^18^ carrier concentration. *ZT* increases with increasing in temperature and pressure. At room temperature, the ZT values of 0.82 and 0.98 K achieved at 22.0 GPa, for n and p-types Tl_2_O_3_, respectively. High pressure as well as temperature have a strong effect on the figure of merit (*ZT*) of Tl_2_O_3_. In Table 1, obtained results are compared with the thermoelectric properties of TCOs. These thermoelectric findings imply that this TCO material can be used in the fabrication of thermoelectric devices under pressure.

## Conclusion

The effect of pressure on the structural, optoelectronic and thermoelectric properties of Tl_2_O_3_ were investigated by using the FP-LAPW method and the modified Becke-Johnson (mBJ) functional with spin–orbit coupling. In band structure, obtained direct band gap of Tl_2_O_3_ is 1.23 eV at zero pressure. The results show that the band gap and the effective mass of carries increases with pressure. The bottom of the conduction band composed of Tl-6s dangle band and the hydrostatic pressure significantly shifts Tl-6s state. By increasing pressure, the blue shift observed in optical responses such as the real and the imaginary parts of the dielectric functions, electron energy loss and absorption spectra. For n- type Tl_2_O_3_, the Seebeck coefficient increases with pressure and reaches to − 292 μV/K at room temperature but thermal conductivity decrease under the effect of pressure. By increasing pressure, it is possible to control the high Seebeck coefficient and the low thermal conductivity for Tl_2_O_3_ compound. The maximum figure of merit *ZT* of p-type Tl_2_O_3_ is 0.98. The results indicate that pressure and temperature tuning play significant roles in the design of thermoelectric devices based on Tl_2_O_3_.

## Data Availability

The data that support the findings of this study are available from the corresponding author H. A. Rahnamaye Aliabad, upon reasonable request.
